# Assessment of the LDL-C/HDL-C ratio as a predictor of one year clinical outcomes in patients with acute coronary syndromes after percutaneous coronary intervention and drug-eluting stent implantation

**DOI:** 10.1186/s12944-019-0979-6

**Published:** 2019-02-02

**Authors:** Zhixiong Zhong, Jingyuan Hou, Qifeng Zhang, Wei Zhong, Bin Li, Cunren Li, Zhidong Liu, Min Yang, Pingsen Zhao

**Affiliations:** 1 Center for Cardiovascular Diseases, Meizhou People’s Hospital (Huangtang Hospital), Meizhou Academy of Medical Sciences, Meizhou Hospital Affiliated to Sun Yat-sen University, Meizhou, 514031 People’s Republic of China; 2 Clinical Core Laboratory, Meizhou People’s Hospital (Huangtang Hospital), Meizhou Academy of Medical Sciences, Meizhou Hospital Affiliated to Sun Yat-sen University, Meizhou, 514031 People’s Republic of China; 3 Center for Precision Medicine, Meizhou People’s Hospital (Huangtang Hospital), Meizhou Academy of Medical Sciences, Meizhou Hospital Affiliated to Sun Yat-sen University, Meizhou, 514031 People’s Republic of China; 4 Guangdong Provincial Engineering and Technological Research Center for Molecular Diagnostics of Cardiovascular Diseases, Meizhou, 514031 People’s Republic of China; 5 Meizhou Municipal Engineering and Technology Research Center for Molecular Diagnostics of Cardiovascular Diseases, Meizhou, 514031 People’s Republic of China; 6 Meizhou Municipal Engineering and Technology Research Center for Molecular Diagnostics of Major Genetic Disorders, Meizhou, 514031 People’s Republic of China; 7 Guangdong Provincial Key Laboratory of Precision Medicine and Clinical Translational Research of Hakka Population, Meizhou, 514031 People’s Republic of China; 8 Guangdong Provincial Engineering and Technology Research Center for Clinical Molecular Diagnostics and Antibody Therapeutics, No 63 Huangtang Road, Meijiang District, Meizhou, 514031 People’s Republic of China

**Keywords:** Acute coronary syndromes, Percutaneous coronary intervention, Drug-eluting stent implantation, Low-density lipoprotein, High-density lipoprotein, Major adverse cardiac events

## Abstract

**Background:**

Despite significant advances in the management of acute coronary syndromes (ACS), there are still plenty of patients undergoing percutaneous coronary intervention (PCI) and stent implantation suffered poor prognosis and high treatment expenditure. Evidence increasingly suggests that the ratio of low-density lipoprotein cholesterol/high-density lipoprotein cholesterol (LDL-C/HDL-C) ratio might be a novel marker for the risk of atherosclerotic cardiovascular disease, but the impact of LDL-C/HDL-C ratio on 1-year prognosis of drug-eluting stent (DES) implantation patients after PCI is still not reported. Our aim of the study was to investigate the impact of LDL-C/HDL-C ratio on 1-year prognosis of DES implantation patients after PCI.

**Methods:**

Between May 2014 and July 2016, 1937 patients who were underwent primary PCI and DES implantation and achieving LDL-C with statins were enrolled and divided into two groups based on the ratio of LDL-C/HDL-C.

**Results:**

The entire occurrence of adverse cardiovascular events according to the ratio of LDL-C/HDL-C showed that there were no significant differences in 1-year cardiovascular death (hazard ratio [HR]: 1.97, 95% confidence interval [CI]: 0.49 to 7.84, *P* = 0.329), myocardial infarction (MI) (HR: 1.66, 95% CI: 0.84 to 3.28, *P* = 0.172) and bleeding events (HR: 1.08, 95% CI: 0.83 to 1.41, *P* = 0.598) The cumulative incidence of target lesion revascularization (TLR) (HR: 1.43, 95% CI: 1.10 to 1.86, *P* = 0.007), stent thrombosis (ST) (HR: 2.04, 95% CI: 1.06 to 3.93, *P* = 0.037) and major adverse cardiac events (MACE) (HR: 1.54, 95% CI: 1.24 to 1.91, *P* <  0.001) were significantly higher in high group than in low group. Multivariate Cox regression analysis revealed that age (HR: 1.556, 95%, CI: 1.198 to 2.021, *P* <  0.001), together with diabetes mellitus (HR: 1.490, 95% CI: 1.142 to 1.945, *P* = 0.003), and ratio of LDL-C/HDL-C (HR: 1.638, 95% CI: 1.260 to 2.218, *P* <  0.001) were independent predictors of 1-year MACE. The Kaplan-Meier cumulative MACE-free survival curves with a log-rank test showed that the presence of high ratio of LDL-C/HDL-C was associated with higher incidences of MACE after PCI with DES implantation.

**Conclusions:**

The high LDL-C/HDL-C ratio was associated with cardiovascular events in patients with ACS after PCI and DES implantation.

## Background

Cardiovascular diseases (CAD) is a significant global health problem and is considered as the leading cause of death worldwide [1]. Currently, approximately 46 million people in China are affected by CAD with the changes lifestyle and longer life spans [2]. With the introduced of coronary artery bypass graft (CABG) surgery and percutaneous coronary intervention (PCI), substantial breakthrough have been made in the treatment of CAD, especially in patients with acute coronary syndromes (ACS) [3, 4]. In particular, the advent of drug-eluting stent (DES) has significantly reduced the occurrence of vessel revascularization and major adverse cardiac events (MACE) [5, 6]. Despite significant advances in the management of ACS, there are still plenty of patients undergoing PCI and stent implantation suffered poor prognosis and high treatment expenditure attribute to the varying disease severity [7, 8]. Therefore, identifying cardiovascular risk factors that affect the prognosis of patients and implementation of treatments to minimize risk of new ischemic events after PCI treatment is necessary [9–12].

Previous literature has illustrated that dyslipidemia is a risk factor for development and progression of coronary arteriosclerosis and cardiovascular outcomes in patients with CAD [13, 14]. Our previous research confirmed that serum lipid levels varied in age and gender in Hakka patients with acute myocardial infarction (AMI). Dyslipidemia is more prevalent in the non-elderly than in the elderly for males. Levels of total cholesterol (TC), triglycerides (TG) and low-density lipoprotein cholesterol (LDL-C) were higher in females than in males for the elderly Hakka population in southern China [15]. Numerous evidences have revealed that LDL-C serum concentrations correlate with very high cardiovascular risk and stringent LDL-C lowering with statins therapy is recommended for the secondary prevention of recurrent cardiovascular events and survival improvement after PCI by the current international guidelines [16, 17]. Another research of ours found that PCI patients with lower preprocedural LDL-C were less risky to periprocedural myocardial injury in southern China [18]. Data from patients treated with statins show considerable gains by approximately 22% per 1.0 mmol/L LDL-C reduction [19]. However, many patients achieving very low levels of LDL-C showing widely different treatment effects and subsequently experienced a cardiovascular event because of the residual risk factors [11, 13]. Recently, serum levels of high-density lipoprotein cholesterol (HDL-C) was recognized significantly and inversely associated with the development of atherosclerotic cardiovascular diseases [20, 21]. Evidence increasingly suggests that the ratio of LDL-C/HDL-C ratio might be a novel marker of the risk of atherosclerotic cardiovascular disease, as it simultaneously evaluates the levels of both LDL-C and HDL-C [22, 23]. However, there is little information regarding the predictive value of the LDL-C/HDL-C ratio for detecting cardiovascular events in patients with ACS after DES implantation and more studies are needed to further characterize the relationship between the LDL-C/HDL-C ratio and clinical outcome. The purpose of this study was to investigate the impact of LDL-C/HDL-C ratio on 1-year prognosis of DES implantation patients after PCI.

## Materials and methods

### Study population

This was a retrospective observational study. Between May 2014 and July 2016, a total of 1937 consecutive participants who had the initiation of statins therapy with atorvastatin after successful primary PCI were enrolled from the patients who had been hospitalized at department of cardiology in the Meizhou People’s Hospital. All patients had measurements of serum lipid profiles at baseline and at 4 weeks after atorvastatin therapy and lipid levels were found to remain constant during 1-year follow up. The study protocol was approved by the Ethics Committee of Meizhou People’s Hospital (Huangtang Hospital), Meizhou Academy of Medical Sciences, Meizhou Hospital Affiliated to Sun Yat-sen University, Guangdong province, China, and conforms to the ethical principles of the Declaration of Helsinki. All participants have signed the written informed consents.

Patients were eligible for enrollment if (1) if they were18 years of age or older; (2) if they were atorvastatin-treated who underwent PCI for symptomatic coronary artery disease and DES implantation. Exclusion criteria were severe liver and kidney dysfunctions, malignant tumors, severe autoimmune diseases. Patients whose lipid levels were found to change dynamically during 1-year follow up were also excluded from the study.

### Sample collection and follow-up

Detailed medical charts were performed of the initial hospitalization to obtain patients’ medical history, laboratory results, and medications. For blood lipid detection, fasting blood samples were collected from each subject and anticoagulated with ethylenediamine tetraacetic acid (EDTA) dipotassium salt in the early morning. The sample was separated immediately by centrifugation at 3000 g for 15 min at 4 °C to retrieve plasma. Lipid profiles were measured enzymatically on a chemistry analyzer (AU5400 analyzer, Beckman Coulter, CA, USA).

Follow-up were scheduled from the date of discharge for the index hospitalization. Follow-up data were collected by telephone interviews and entered into a computer database by trained staff and by inviting patients or their relatives to complete a standardized questionnaire which was sent to the patients during the follow-up collection period. The preferred definition of MACE was the composite of cardiovascular death, nonfatal myocardial infarction (MI), and target lesion revascularization (TLR), stent thrombosis (ST). All-cause death was defined as any death during or after the procedure and was considered to be of cardiac origin unless a definite noncardiac cause could be established. MI was defined as a recent ischaemic symptom with new electrocardiographic changes and a positive troponin concentration exceed the upper limit of normal. TLR was defined as repeated revascularization of the target vessel by PCI or surgical bypass of any vessel. ST and bleeding events were assessed according to the Bleeding Academic Research Consortium classification [24].

### Statistical analysis

All statistical analyses were performed with the SPSS 19.0 statistical package program (SPSS, Chicago, IL, USA). Continuous variables were expressed as mean ± standard deviation (SD) and categorical variables as percentage. Analysis of variance and the chi-square test were used for continuous and categorical variables, respectively. Multivariate Cox regression analysis using the entered method was used to evaluate the independent predictors of clinical endpoint. MACE event-free rates for clinical outcomes were constructed using the Kaplan-Meier method and compared with the log-rank test. A *P* <  0.05 was recognized as statistically significant.

## Results

### Patient characteristics

**Fig. 1:**
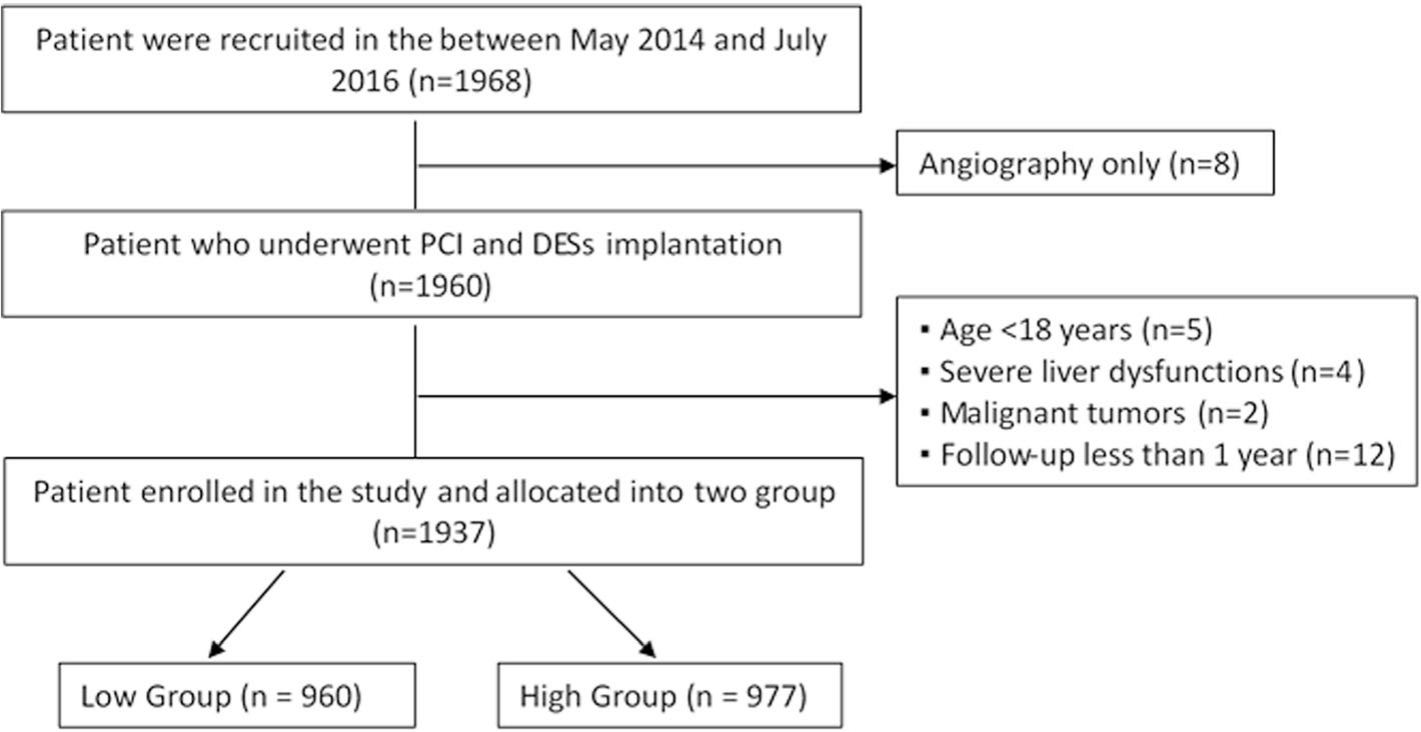
Flow chart of patient enrollment

A flow chart of the patient enrollment was provided as Fig. 1. Subsequent to coronary angiography, a total of 1968 consecutive ACS patients who underwent PCI with successful DES implantation and cholesterol-lowering therapy with atorvastatin were enrolled, of whom 1937 eligible patients were included in this analysis. Patients were allocated into low and high ratio groups according to the median value of the LDL-C/HDL-C ratio (the median value was 2.7) in each subgroup of study patients. The baseline clinical and demographic characteristics of the recruited patients were detailed in Table 1. The patients in low group were older than those of the high group. There was a higher proportion of ST-elevation myocardial infarction (STEMI) in low group compared to the high group. There were statistically significant differences in term of dyslipidemia, TG, LDL-C, HDL-C, hemoglobin and platelet. No significant differences were observed for other variables between the two groups.

**Table 1:** Baseline characteristics of the study cohorts

Characteristics	Overall (*n* = 1937)	Low	High	*P* value
		(*n* = 960)	(*n* = 977)	
Age, years	64.0 ± 10.8	65.0 ± 10.7	63.0 ± 10.8	< 0.001
Male, n(%)	1475 (76.1)	702 (73.1)	773 (79.1)	0.002
Smoking, n(%)	738 (38.1)	361 (37.6)	377 (38.6)	0.656
Drinking, n(%)	93 (4.8)	57 (5.9)	42 (4.3)	0.121
Systolic BP, mmHg	134.0 ± 24.6	134.6 ± 24.6	133.6 ± 24.7	0.400
Previous MI, n(%)	50 (2.6)	30 (3.1)	19 (1.9)	0.098
Hypertension, n(%)	1066 (55.0)	523 (54.5)	543 (55.6)	0.627
Dyslipidemia, n(%)	704 (36.3)	254 (26.5)	450 (46.1)	< 0.001
Diabetes mellitus, n(%)	552 (28.5)	262 (27.3)	290 (29.7)	0.244
LV ejection fraction, (%)	56.3 ± 9.6	56.4 ± 9.2	56.3 ± 10.0	0.836
Clinical presentation				
Unstable angina, n(%)	598 (30.9)	277 (28.9)	321 (32.9)	0.062
NSTEMI, n(%)	320 (16.5)	144 (15.0)	176 (18.0)	0.076
STEMI, n(%)	1022 (52.8)	542 (56.5)	480 (49.1)	0.001
Number of vessels				
Single, n(%)	1119 (57.8)	539 (56.1)	580 (59.4)	0.154
Double, n(%)	473 (24.4)	243 (25.3)	230 (23.5)	0.369
Triple, n(%)	345 (17.8)	178 (18.5)	167 (17.1)	0.407
Stent diameter, mm	3.13 ± 0.45	3.13 ± 0.44	3.13 ± 0.46	0.833
Stent length, mm	34.87 ± 13.88	35.45 ± 13.22	34.31 ± 14.46	0.070
Medications				
Aspirin	1737 (89.7)	868 (90.4)	869 (88.9)	0.297
Clopidogrel	1728 (89.2)	868 (90.4)	860 (88.0)	0.093
ACEI/ARB, n(%)	1597 (82.4)	787 (82.0)	810 (82.9)	0.592
β-blocker, n(%)	1603 (82.8)	782 (81.5)	821 (84.0)	0.149
PPI, n(%)	1351 (68.7)	686 (71.5)	665 (68.1)	0.113
Calcium blockers, n(%)	269 (13.9)	132 (13.8)	137 (14.0)	0.862
Laboratory parameters				
TC, mmol/L	1.96 ± 1.50	1.95 ± 1.84	1.97 ± 1.06	0.751
TG, mmol/L	4.94 ± 1.23	4.55 ± 1.22	5.31 ± 1.24	< 0.001
LDL-C, rmmol/L	3.07 ± 0.97	2.49 ± 0.67	3.65 ± 0.88	< 0.001
HDL-C, mmol/L	1.18 ± 0.42	1.32 ± 0.52	1.04 ± 0.21	< 0.001
Hemoglobin, g/L	134.80 ± 27.66	131.20 ± 19.01	138.30 ± 33.73	< 0.001
Platelet, ×10^9^/L	232.00 ± 75.52	226.10 ± 79.21	237.80 ± 71.28	< 0.001

*BP* Blood pressure, *LV* Left ventricular, *NSTEMI* Non-ST elevation myocardial infarction, *STEMI* ST elevation myocardial infarction, *LAD* Left anterior descending artery, *LCX* Left circumflex artery, *RCA* Right coronary artery, *ACEI* angiotensin-converting enzymeinhibitor, *ARB* Angiotensin receptor blocker, *PPI* Proton-pump inhibitor, *TC* Total cholesterol, *TG* Triglycerides, *LDL-C* Low-density lipoprotein cholesterol, *HDL-C* High-density lipoprotein cholesterol

Data presented are mean ± SD or n(%)

### Clinical outcomes

**Fig. 2:**
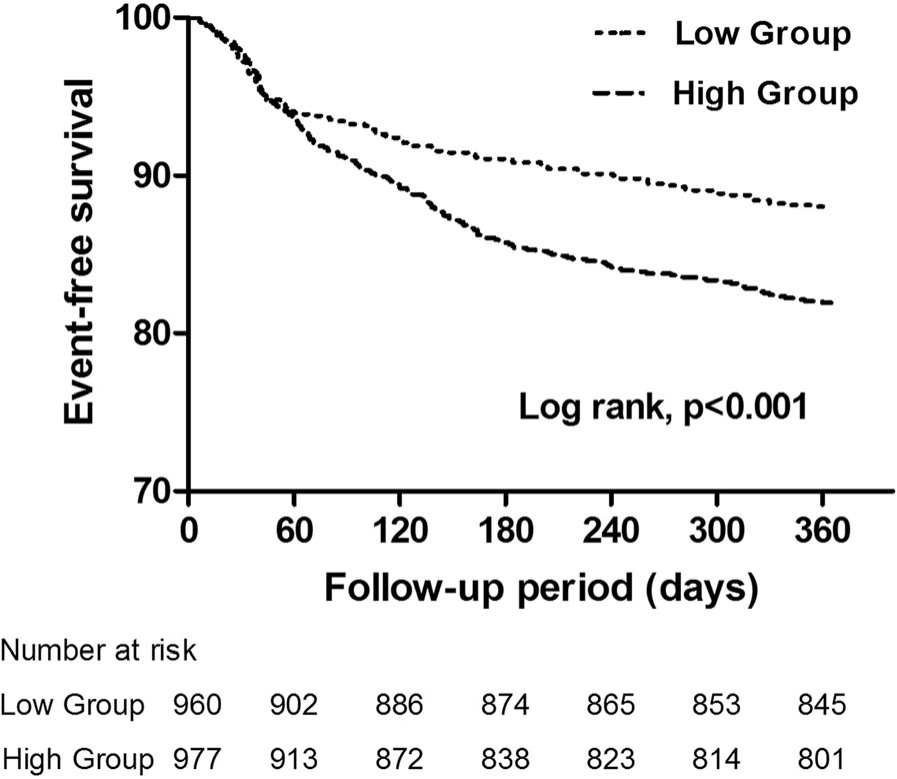
Kaplan-Meier curves for 1-year cardiovascular events

Table 2 shows the entire occurrence of adverse cardiovascular events according to the ratio of LDL-C/HDL-C. We found that there were no significant differences in 1-year cardiovascular death (hazard ratio [HR]: 1.97, 95% confidence interval [CI]: 0.49 to 7.84, *P* = 0.329), MI (HR: 1.66, 95% CI: 0.84 to 3.28, *P* = 0.172) and bleeding events (HR: 1.08, 95% CI: 0.83 to 1.41, *P* = 0.598). The cumulative incidence of TLR (HR: 1.43, 95% CI: 1.10 to 1.86, *P* = 0.007), ST (HR: 2.04, 95% CI: 1.06 to 3.93, *P* = 0.0368) and MACE (HR: 1.54, 95% CI: 1.24 to 1.91, *P* <  0.001) were significantly higher in high group than in low group. Multivariate Cox regression analysis of the association between the MACE and multiple parameters are presented in Table 3. The results of the multivariable Cox proportional hazards model demonstrated that age (HR: 1.556, 95%, CI:1.198 to 2.021, *P* <  0.001), together with diabetes mellitus (HR: 1.490, 95% CI:1.142 to 1.945, *P* = 0.003), and ratio of LDL-C/HDL-C (HR: 1.638, 95% CI:1.260 to 2.218, *P* <  0.001) were independent predictors of 1-year MACE. Detailed results of the multivariable analysis were shown in Table 3. The Kaplan-Meier cumulative MACE-free survival curves with a log-rank test showed that the presence of high ratio of LDL-C/HDL-C was associated with higher incidences of MACE after PCI with DES implantation (Fig. 2).

**Table 2:** Clinical outcome up to 1-year

	Low (n = 960)	High (n = 977)	Hazard ratio (95% CI)	*P* value
Death	5 (0.52)	8 (0.82)	1.57 (0.52 to 4.79)	0.580
Cardiovascular death	3 (0.31)	6 (0.61)	1.97 (0.49 to 7.84)	0.329
Myocardial infarction	13 (1.35)	22 (2.25)	1.66 (0.84 to 3.28)	0.172
STEMI	8 (0.83)	19 (1.94)	2.33 (1.03 to 5.31)	0.051
NSTEMI	3 (0.31)	3 (0.31)	0.98 (0.20 to 4.86)	1.000
TLR	85 (8.86)	124 (12.69)	1.43 (1.10 to 1.86)	0.007
Stent thrombosis	13 (1.35)	27 (2.76)	2.04 (1.06 to 3.93)	0.037
MACE	114 (11.88)	179 (18.32)	1.54 (1.24 to 1.91)	< 0.001
Bleeding events	93 (9.69)	102 (10.44)	1.08 (0.83 to 1.41)	0.598

*CI* Confidence interval, *NSTEMI* Non-ST elevation myocardial infarction, *STEMI* ST elevation myocardial infarction, *TLR* Target lesion revascularization, *MACE* Major adverse cardiac events; Other abbreviations as in Table 1

Data are presented as n (%)

**Table 3:** Results of a multivariable Cox proportional hazards model

Variable	Hazard ratio (95% CI)	*P* value
Age (age ≥ 65 vs < 65)	1.556 (1.198 to 2.021)	0.001
Gender (male vs female)	0.958 (0.696 to 1.317)	0.790
Smoking (active smoker vs non-smoker)	0.813 (0.603 to 1.098)	0.177
Drinking (drinker vs non-drinker)	0.944 (0.486 to 1.833)	0.865
Hypertension (hypertensive vs normotensive)	0.997 (0.771 to 1.289)	0.981
Diabetes mellitus (diabetic vs non-diabetic)	1.490 (1.142 to 1.945)	0.003
LDL-C/HDL-C (Ratio ≥ 2.7 vs < 2.7)	1.638 (1.260 to 2.128)	< 0.001

## Discussion

To the best of our knowledge, this is the first study reporting regards the associations of LDL-C/HDL-C ratio with the incidences of 1-year adverse outcomes of ACS patients treated with atorvastatin after PCI and DES implantation in Chinese population. In the present study, we found that the rate of TLR, ST and MACE in patients with the high LDL-C/HDL-C ratio was significantly greater than those with the low LDL-C/HDL-C ratio at 1-year follow-up. The result of multivariate analysis showed that older age, diabetes mellitus and the high ratio of LDL-C/HDL-C factors were positively associated with the accumulated MACE during 1-year follow-up. The results presented here indicate that of LDL-C/HDL-C ratio was predictor of MACE at one year in patients with ACS who underwent PCI and DES.

ACS is one of leading cause of death in developed and developing countries. PCI and the application of stents resulted in tremendous progress on the management of patients with ACS, which dramatically reduces the cardiovascular mortality and disability rates [4, 6]. ST and other adverse cardiovascular events, including death, MI, TLR and bleeding events, are considered as life-threatening complications of PCI [25]. Dual antiplatelet therapy consisting of aspirin and clopidogrel is the standard therapy choice for patients with ACS after PCI. However, some patients on standard dual antiplatelet therapy still have at risk of adverse cardiovascular events after DES implantation due to the existence of high platelet reactivity, genetic variation with drug response, older age and dyslipidemia [7, 8, 13].

Numerous of evidences have already revealed that LDL-C serum concentrations is associated with cardiovascular risk and intensive treatment with statin is recommended to reduce the rate of recurrent ischaemic events and stent thrombosis in patients with ACS [13, 26]. Every 1.0 mmoL/L reduction in LDL-C is associated with a corresponding 20–25% reduction in cardiovascular mortality and non-fatal myocardial infarction. According to the current European guidelines, as well as the US guidelines, a treatment goal of LDL-C < 70 mg/dL is recommended [16, 17]. In spite of the emphasis of guidelines on the tight control of the LDL-C level, several surveys have shown that still a large number of patients remain undertreated and do not attain LDL-C treatment goals [11, 12, 27]. Similarly, our result is in line with that reported in previous studies.

It is also important to note that mixed dyslipidaemia played an important role in the propagation of coronary artery disease [28]. Epidemiological data have demonstrated that low levels of high-density lipoprotein cholesterol (HDL-C) was an important risk factor for progression of coronary atherosclerosis and moderate increases in HDL-C in statin-treated patients are correlated with regression of coronary atherosclerosis [20, 29, 30]. It has been also suggested that HDL-C could reduce the risk and extent of PCI-related myocardial infarction by stabilizing plaques. However, a paradoxical decrease in HDL-C levels after statin therapy is often seen in clinical settings. Previous lipid-lowering trial have shown that the stable lipid level after statin treatment is achieved after 4–6 weeks, and then the lowered lipid level remained during the subsequent maintenance treatment [26, 31]. Take these things together, the ratio of LDL-C/HDL-C ratio might be the useful indicators for prognosis prediction as it simultaneously evaluates the levels of both LDL-C and HDL-C. Some recent studies have considered the LDL-C/HDL-C ratio to be a sensitive marker of the risk of atherosclerotic cardiovascular disease, as it simultaneously evaluates the levels of both LDL-C and HDL-C [32, 33]. Several studies have previously demonstrated the LDL-C/HDL-C ratio is an important contributor to the development of new coronary artery disease in patients with a previous history of PCI [22]. Moreover, a prior report has found that the LDL-C/HDL-C ratios is a useful indicator for preventing the progression of atherosclerotic cardiovascular disease [34]. In the present study, our results indicated that the high LDL-C/HDL-C ratio group was at a high risk of 1-year adverse outcomes in patients with ACS after PCI and DES implantation. In addition, several factors including advanced age, diabetes mellitus and major organ dysfunction are well-established predictors of worse clinical outcomes in patients with ACS or PCI [9, 35, 36]. In current study, the multivariable Cox hazards analysis of MACE in our study revealed that older age, diabetes mellitus and the high ratio of LDL-C/HDL-C were independent predictors for the MACE at 1-year follow up. Moreover, the Kaplan-Meier cumulative MACE-free survival curves with a log-rank test also showed that the presence of high ratio of LDL-C/HDL-C might serve as a predictor of one year clinical outcomes in patients with ACS after PCI and DES implantation. Our results are consistent with the findings from previous study.

Our study has several limitations. First, a major limitation is its retrospective design and was subject to bias. Second, as this is a single center study and the sample size was relatively small, the evidence may not be as persuasive as that obtained by a larger scale and multiple regions. Third, the follow-up period was relatively short, a long-term prospective large-scale study is needed.

## Conclusions

In conclusion, the results of this study found that the high LDL-C/HDL-C ratio was associated with cardiovascular events in patients with ACS after PCI and DES implantation. For patients with ACS after DES implantation, LDL-C/HDL-C ratio may help in planning more aggressive cholesterol-lowering therapy to improve the clinical outcome. Further large prospective studies are needed to confirm and to reveal clinical implications of our findings.

## Abbreviations

ACEI: Angiotensin-converting enzymeinhibitor
ACS: Acute coronary syndromes
AMI: Acute myocardial infarction
ARB: Angiotensin receptor blocker
BP: Blood pressure
CABG: Coronary artery bypass graft
CAD: Cardiovascular diseases
CI: Confidence interval
DES: Drug-eluting stents
EDTA: Ethylenediamine tetraacetic acid
HDL-C: High-density lipoprotein cholesterol
HR: Hazard ratio
LAD: Left anterior descending artery
LCX: Left circumflex artery
LDL-C: Low-density lipoprotein cholesterol
LV: Left ventricular
MACE: Major adverse cardiac events
NSTEMI: Non-ST elevation myocardial infarction
PCI: Percutaneous coronary intervention
PPI: Proton-pump inhibitor
RCA: Right coronary artery
SD: Standard deviation
ST: Stent thrombosis
STEMI: ST elevation myocardial infarction
TC: Total cholesterol
TG: Triglycerides
TLR: Target lesion revascularization
